# Involvement of the Na^+^, K^+^-ATPase α1 Isoform and Endogenous Cardiac Steroids in Depression- and Manic-like Behaviors

**DOI:** 10.3390/ijms25031644

**Published:** 2024-01-29

**Authors:** Noa Horesh, Ilana Pelov, Ilana Pogodin, Hiba Zannadeh, Haim Rosen, Anastasiia Leonidovna Mikhrina, Moran Dvela-Levitt, Vishnu Priya Sampath, David Lichtstein

**Affiliations:** 1 Department of Medical Neurobiology, Institute for Medical Research Israel-Canada, Faculty of Medicine, The Hebrew University of Jerusalem, Jerusalem 91905, Israel; noa.rosenthal1@mail.huji.ac.il (N.H.); ilana.pogodin@mail.huji.ac.il (I.P.); hiba.hani1999@gmail.com (H.Z.); mihrinamail@gmail.com (A.L.M.); vishnulifebt@yahoo.com (V.P.S.); 2 Jerusalem Mental Health Center, Eitanim Psychiatric Hospital, Jerusalem 91060, Israel; ilonaf@gmail.com; 3 Department of Microbiology and Molecular Genetics, Institute for Medical Research Israel-Canada, Faculty of Medicine, The Hebrew University of Jerusalem, Jerusalem 91905, Israel; haimr@ekmd.huji.ac.il; 4 The Mina and Everard Goodman Faculty of Life Sciences, Bar-Ilan University, Ramat-Gan 52900, Israel; dvelamo@gmail.com

**Keywords:** bipolar disorder, Na^+^, K^+^-ATPase, α isoform, intracellular signaling, endogenous cardiac steroids, forced swimming test, open field test

## Abstract

Bipolar disorder (BD) is a severe and common chronic mental illness characterized by recurrent mood swings between depression and mania. The biological basis of the disease is poorly understood, and its treatment is unsatisfactory. Na^+^, K^+^-ATPase is a major plasma membrane transporter and signal transducer. The catalytic α subunit of this enzyme is the binding site for cardiac steroids. Three α isoforms of the Na^+^, K^+^-ATPase are present in the brain. Previous studies have supported the involvement of the Na^+^, K^+^-ATPase and endogenous cardiac steroids (ECS) in the etiology of BD. Decreased brain ECS has been found to elicit anti-manic and anti-depressive-like behaviors in mice and rats. However, the identity of the specific α isoform involved in these behavioral effects is unknown. Here, we demonstrated that decreasing ECS through intracerebroventricular (i.c.v.) administration of anti-ouabain antibodies (anti-Ou-Ab) decreased the activity of α1^+/−^ mice in forced swimming tests but did not change the activity in wild type (wt) mice. This treatment also affected exploratory and anxiety behaviors in α1^+/−^ but not wt mice, as measured in open field tests. The i.c.v. administration of anti-Ou-Ab decreased brain ECS and increased brain Na^+^, K^+^-ATPase activity in wt and α1^+/−^ mice. The serum ECS was lower in α1^+/−^ than wt mice. In addition, a study in human participants demonstrated that serum ECS significantly decreased after treatment. These results suggest that the Na^+^, K^+^-ATPase α1 isoform is involved in depressive- and manic-like behaviors and support that the Na^+^, K^+^-ATPase/ECS system participates in the etiology of BD.

## 1. Introduction

Bipolar disorder (BD) is one of the most impairing psychiatric illnesses. Although the etiology of BD remains to be elucidated, the disease has a strong genetic component, as evidenced by the many genes that have been associated with this condition [1,2].

The Na^+^, K^+^-ATPase is an enzyme present in the plasma membranes of most eukaryotic cells, hydrolyses ATP, and uses the free energy to drive the transport of potassium into the cell and sodium out of the cell, against their electrochemical gradients. This pump function is a key contributor to the asymmetrical distribution of Na^+^ and K^+^ ions and the resting membrane potential; moreover, it accounts for typically 30% and up to 70% in nerve cells of the cellular ATP expenditure. The pump has important roles in regulating cell volume, cytoplasmic pH, and Ca^++^ levels (through the sodium–proton and sodium–calcium exchangers, respectively) and in driving a variety of secondary transport processes [3,4]. The Na^+^, K^+^-ATPase is a hetero-oligomer composed of stoichiometric amounts of two major polypeptides, the α and the β-subunits [5]. Four genes encode the α-subunits α1, α2, α3, and α4, and four genes encode the four β isoforms β1, β2, β3, and β4 [6,7]. Both the α and β-isoforms exhibit species-, tissue-, and cell-specific patterns of expression. The α1 isoform is ubiquitously expressed in the CNS; the α2 isoform is expressed predominantly in astrocytes and other glial cells; and the α3 isoform is expressed primarily in neurons, ovaries, and white blood cells [3,8]. Beyond its roles in ion transport, the Na^+^, K^+^-ATPase engages in signaling to different intracellular compartments. The binding of cardiac steroids at low concentrations to Na^+^, K^+^-ATPase results in the activation of various signal transduction cascades, such as the PI3K1A/Akt; IP3R/NF-κB and RAS-Raf-Erk1/2 pathways, and changes in the levels of reactive oxygen species and intracellular Ca^2+^ [9,10].

Cardenolides (e.g., ouabain and digoxin) and bufadienolides (e.g., bufalin and marinobufagenin) have been used for many years and are currently being used to treat cardiac failure and arrhythmias in western and eastern medicine [11,12]. Compounds identical or similar to cardiac steroids have been identified in mammalian tissues. These compounds include ouabain [13], digoxin [14], and several bufadienolide-like compounds [15,16]. These endogenous cardiac steroids (ECS) are considered a hormone family involved in numerous physiological processes and pathological states, including salt homeostasis and regulation of blood pressure, cell growth, differentiation, and behavior [17,18,19]. To date, most studies on ECS have examined endogenous ouabain. On the basis of its immunoreactivity with anti-ouabain antibodies (anti-Ou-Ab), this compound has been shown to be present in the mammalian brain and CSF and is considered a potential neuromodulator.

A vast number of studies utilizing genetic, molecular, behavioral, and pharmacological tools have provided strong evidence of the involvement of the Na^+^, K^+^-ATPase/ECS system in BD [20,21,22]. An allelic association between BD and a Na^+^, K^+^-ATPase α subunit gene (ATP1A3) has been reported [23]. Furthermore, a significant association of six single SNPs in the three genes of the Na^+^, K^+^-ATPase α isoforms with BD was demonstrated, thus suggesting that this pump may play a role in the etiology of the disease [24]. BD has consistently been associated with abnormalities in Na^+^, K^+^-ATPase activity in erythrocytes [25], and Na^+^, K^+^-ATPase density is significantly lower in patients with BD than in those with major depression and schizophrenia [26]. The plasma levels of ECS are significantly lower in individuals with mania than in unaffected controls [27], whereas the levels of these compounds have been found to be higher in the parietal cortex of post mortem samples in individuals with BD than in individuals with schizophrenia or major depression or unaffected individuals [26]. Intracerebroventricular (i.c.v.) administration of ouabain induces hyperactivity in rats [28,29]. The i.c.v. administration of anti-Ou-Ab, which lowers brain ECS, had anti-depressive-like effects, as measured in the forced swimming test (FST) in normal rats [26]. Furthermore, decreasing brain ECS through i.c.v. administration of anti-Ou-Ab has been found to result in anti-manic-like behavior, through prevention of amphetamine-induced hyperactivity [30] in BALB/c and Black Swiss male mice. On the basis of these and other observations, a model for the involvement of the Na^+^, K^+^-ATPase/ECS in BD has recently been presented [22].

Despite this intensive research on the possible involvement of Na^+^, K^+^-ATPase and ECS in behavior, the identity of the specific Na^+^, K^+^-ATPase α isoform participating in ECS-induced behavior is unknown. To address this knowledge gap, we compared the depressive- and manic-like behaviors in the FST and open field test (OFT), respectively, in wild type (wt) and α1 haploinsufficient mice. The effects of decreasing ECS by i.c.v. administration of anti-Ou-Ab on behavior revealed differences between wt and α1^+/−^ mice. Furthermore, we assessed whether circulating ECS might be altered in patients with BD by measuring ECS levels in sera before and after treatment. Our results support the participation of the Na^+^, K^+^-ATPase/ECS system in BD and suggest that the α1 isoform is involved in the ECS-induced changes in behavior.

## 2. Results

### 2.1. Decreased Brain ECS Levels Increase Depressive-like Behavior in C57Bl/6 α1^+/−^ Mice

**Figure 1:**
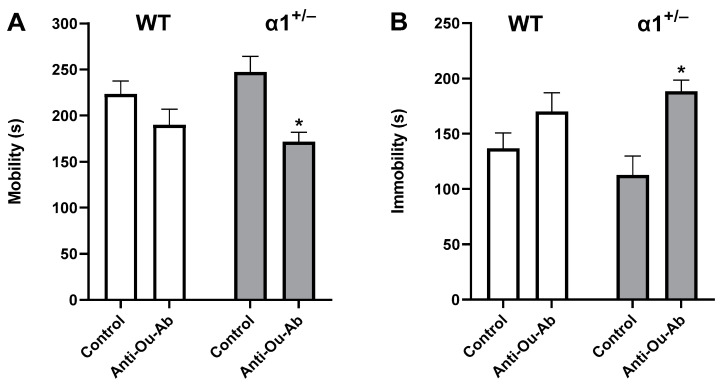
Effects of anti-Ou-Ab administration on behavior in FST in wt and α1^+/−^ mice. Mice received (i.c.v.) either IgG (1 μg/kg, n = 11–14) or anti-ouabain antibodies (1 μg/kg, n = 12–13). Behavior parameters (mobility (**A**) and immobility (**B**)) were monitored during 6-min sessions. Comparisons between groups were analyzed with two-way ANOVA, multiple comparisons test. Values are expressed as mean ± SE (error bars). * *p* < 0.01. vs. α1^+/−^ control.

The behavior of C57Bl/6 wt and α1^+/−^ mice was first examined in the FST, a model of depression-like behavior. As shown in Figure 1A, we did not find a statistically significant difference in mobility between the strains of mice under control conditions. However, after i.c.v. administration of anti-Ou-Ab, we observed a significant decrease in mobility in α1^+/−^ mice (30.57%, Figure 1A). Reciprocal results were obtained in the immobility measurements of mouse behavior, in which treatment with anti-Ou-Ab increased immobility in α1^+/−^ mice (Figure 1B).

### 2.2. Decreased Brain ECS Levels Affect Exploratory and Anxiety Behaviors in α1^+/−^ but Not wt C57Bl/6 Mice

To explore the involvement of ECS in behavior, we used OFT to test the behavioral effect of administration of anti-Ou-Ab into the brains of amphetamine (AMPH)-treated wt and α1^+/−^ mice. OFT is a simple sensorimotor test used to determine general activity levels, gross locomotor activity, and exploration habits in rodent models of CNS disorders. The motor activities (distance moved, duration, and velocity of movement) are considered parameters of depression- or mania-like behaviors, and the number of entries and time spent in the center or periphery are indicators of anxiety and exploration behaviors, respectively [31,32].

**Figure 2:**
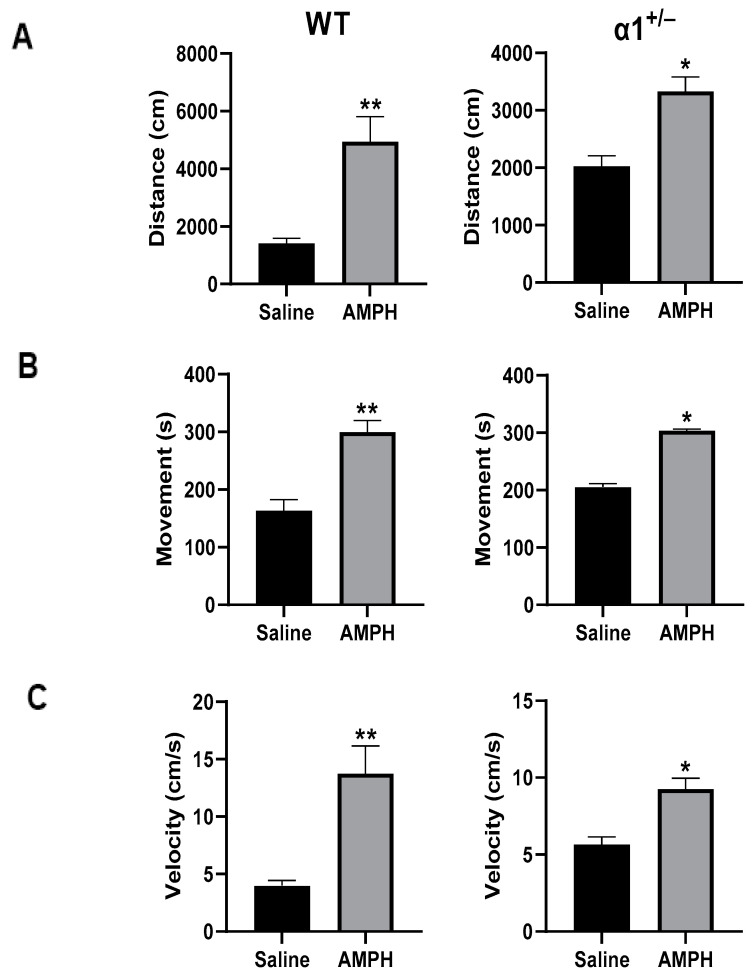
Effects of AMPH treatment (i.p.) on behavior in OFT in wt and α1^+/−^ mice. The two strains of mice received AMPH (5 mg/kg, i.p., n = 8). The mice were placed in an open field within 15 min after treatment, and their behavior was monitored. Three parameters were tested: distance moved (cm) (**A**), duration of mobility (s) (**B**), and mean velocity (cm/s) (**C**) during a 6-min session. An unpaired two-tailed T-test was performed. Values are expressed as mean ± SE (error bars). * *p* < 0.05, vs. respective control, ** *p* < 0.001 vs. respective control.

**Figure 3:**
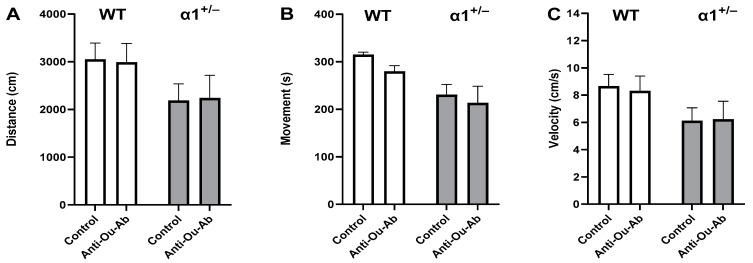
Effects of anti-Ou-Ab administration on AMPH-induced hyperactivity in wt and α1^+/−^ mice. The two strains of mice, wt and α1^+/−^, received either IgG (1 μg/kg, i.c.v., n = 11–12, control) or anti-ouabain antibodies (1 μg/kg, i.c.v., n = 8–17, anti-Ou-Ab) followed by AMPH injection (5 mg/kg, i.p.). Behavior activity parameters, distance moved (cm) (**A**), duration of mobility (s) (**B**), and mean velocity (cm/s) (**C**) were monitored in an open field for a 6-min session. Differences in behavior were analyzed with multiple comparisons two-way ANOVA. Values are expressed as mean ± SE (error bars).

**Figure 4:**
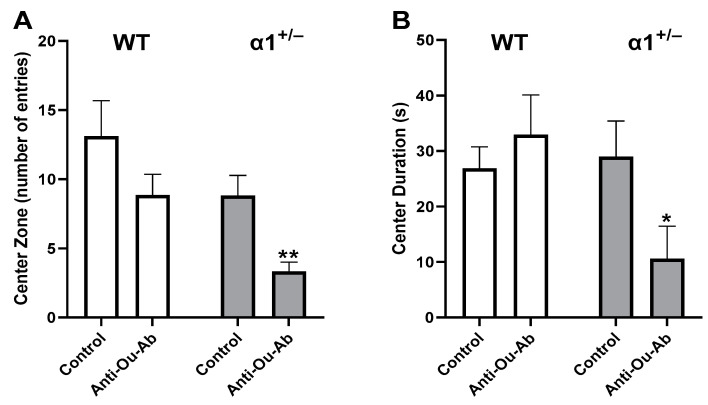

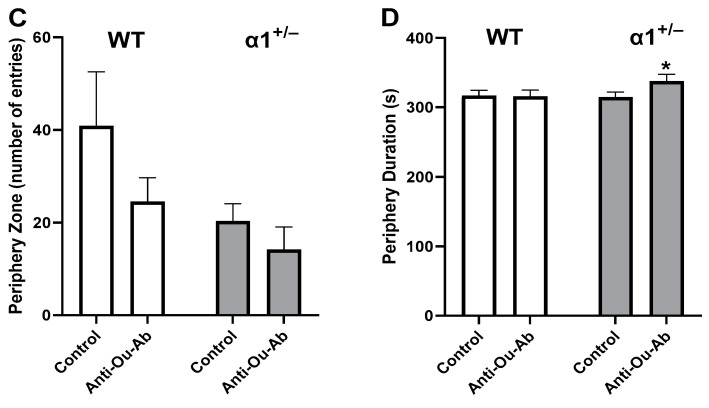
Effects of anti-Ou-Ab administration on AMPH-induced center zone duration and entry in wt and α1^+/−^ mice. The two strains of mice, wt and α1^+/−^, received either IgG (1 μg/kg, i.c.v., n = 11–12, control) or anti-ouabain antibodies (1 μg/kg, i.c.v., n = 8–17, anti-Ou-Ab) followed by AMPH injection (5 mg/kg, i.p.). Behavior parameters, center zone entry and duration (**A**,**B**), and periphery entry and duration (**C**,**D**) were monitored in an open field for a 6-min session. Differences in behavior were analyzed with multiple comparisons two-way ANOVA. Values are expressed as mean ± SE (error bars). * *p* < 0.05 vs. respective control, ** *p* < 0.005 vs. respective controls.

The administration of AMPH increased mobility in wt mice in the OFT, manifesting as increases of 246%, 83%, and 246% in the distance moved (Figure 2A), the duration of mobility (Figure 2B), and the mean velocity (Figure 2C), respectively. Similarly, treatment of α1^+/−^ mice with AMPH increased all measured parameters in the OFT (Figure 2A–C). Mice that received injection of anti-Ou-Ab into the brain showed different response to AMPH treatment: the administration of anti-Ou-Ab into the lateral ventricle immediately after AMPH intraperitoneal (i.p.) administration did not affect AMPH-induced hyperactivity in wt and α1^+/−^ mice. This is based on the absence of changes in measured distance moved (Figure 3A), duration of mobility (Figure 3B), and velocity (Figure 3C) under all experimental conditions. However, this treatment decreased the number of entries into the center zone by 62% (Figure 4A) and increased the periphery duration time by 7% (Figure 4B) in α1^+/−^ but not wt mice, thereby indicating increased anxiety after decreased ECS in the haploinsufficient mice. In addition, this treatment decreased center duration in the α1^+/−^ mice by 63%, thus indicating decreased exploratory behavior, but did not have similar effects in wt mice (Figure 4C). These findings suggest that the α1 isoform is involved in the ECS-induced changes in behavior (described in the Discussion).

### 2.3. ECS Levels and Na^+^, K^+^-ATPase Activity in the Brain in wt and α1^+/−^ Mice

Since i.c.v. administration of anti-Ou-Ab affected behavior in the FST in α1^+/−^ mice, we sought to determine ECS levels and Na^+^, K^+^-ATPase activity in the brain in this experimental group.

**Figure 5:**
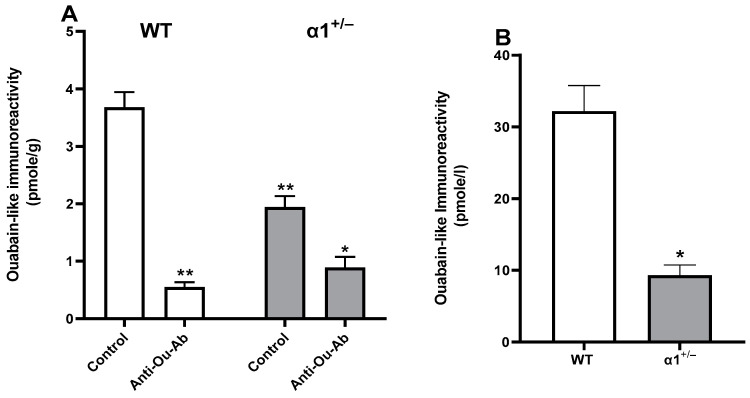
Effects of anti-Ou-Ab administration on ECS (measured as ouabain-like immunoreactivity) levels in the brains and serum of wt and α1^+/−^ mice. Wt and α1^+/−^ mice were sacrificed immediately after the FST (Figure 1). The brain cortex was excised and extracted, and ECS levels were determined with enzyme-linked immunosorbent assays (Section 4) (**A**). Serum samples were collected from the mice and ECS were extracted and determined (**B**). Differences in steroid levels were analyzed with a multiple comparisons two-way ANOVA. Values are expressed as mean ± SE (error bars). * *p* < 0.05 vs. respective control, ** *p* < 0.005 vs. respective controls.

The levels of ECS in the α1^+/−^ mice were significantly lower than those in wt mice (1.94 ± 0.43 pmol/g and 3.68 ± 0.65, respectively) (Figure 5). Furthermore, after administration of anti-Ou-Ab, the levels of ECS declined dramatically, by 54% and 85% in the wt and α1^+/−^ mice, respectively (Figure 5).

**Figure 6:**
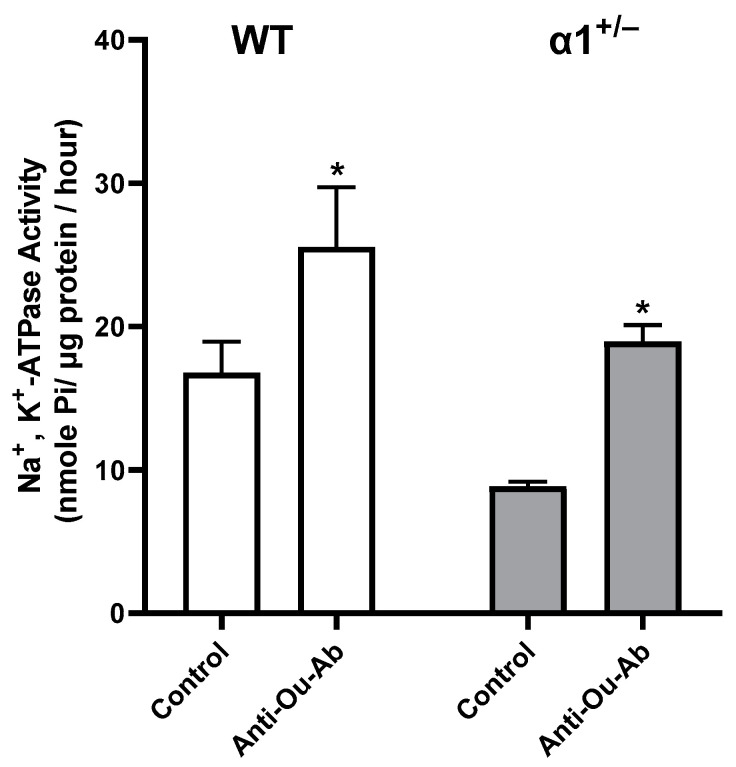
Effects of anti-Ou-Ab administration on Na^+^, K^+^-ATPase activity in the brains of wt and α1^+/−^ mice. Wt and α1^+/−^ mice were sacrificed immediately after the FST experiments (Figure 1). Mouse brain cortex was excised and used for the preparation of plasma membrane synaptosomal fraction (Section 4). Na^+^, K^+^-ATPase activity in the synaptosomal fraction was determined as previously described (Section 4). Na^+^, K^+^-ATPase activity, expressed in nmol Pi/µg protein/h, represented more than 80% of the total ATPase activity in the preparation. Differences in enzymatic activity were analyzed with a multiple comparisons two-way ANOVA. Values are expressed as mean ± SE (error bars). * *p* < 0.05 vs. respective controls.

The effect of the administration of anti-Ou-Ab on Na^+^, K^+^-ATPase activity in wt and α1^+/−^ mice in this experimental group (after FST) is presented in Figure 6. Statistically significant 114% and 52% increases in activity were observed in the α1^+/−^ and wt mice, respectively.

### 2.4. Decreased Serum ECS in Patients with BD after Treatment

**Figure 7:**
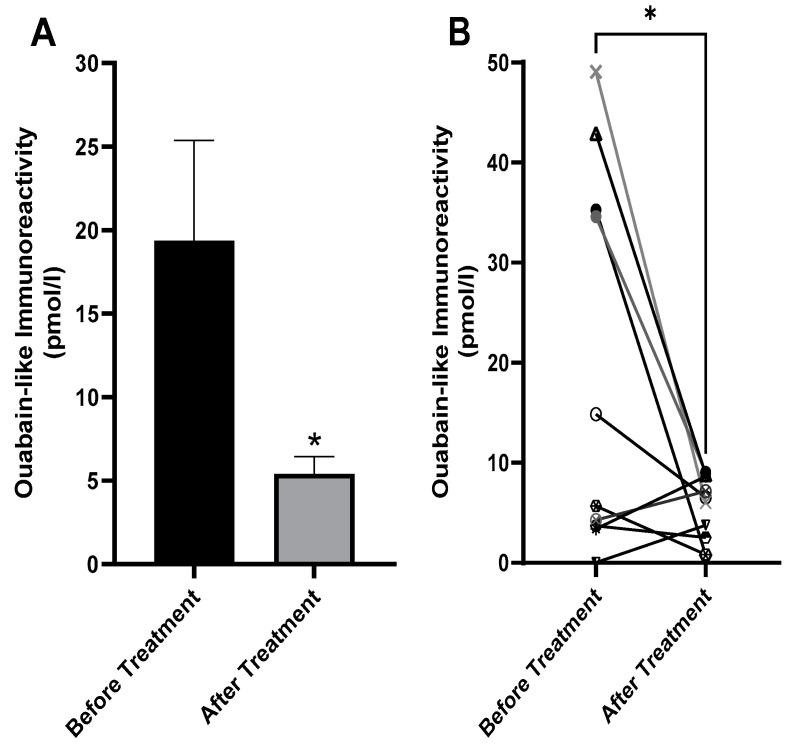
Effects of treatment on the levels of ECS (measured as ouabain-like immunoreactivity) in the serum of bipolar patients. Blood samples were collected from ten patients immediately after admission to the acute psychiatric unit of the Eitanim Psychiatric Hospital because of a manic psychotic episode. A second blood sample was collected 2–3 weeks later, after the patients had stabilized. The serum was extracted, and the levels of ECS were determined (Section 4). The average ECS levels before and after treatment are depicted in (**A**). The change in ECS in each patient is shown in (**B**). The differences in steroid levels before and after treatment were analyzed with nonparametric paired *t* tests (Wilcoxon). Values are expressed as mean ± SE (error bars). * *p* <0.05 vs. after treatment.

The experiments in the above animal models suggested that the interaction of ECS with the Na^+^, K^+^-ATPase α1 isoform is involved in depressive-like behavior, in agreement with our hypothesis that the Na^+^, K^+^-ATPase/ECS system participates in BD [22]. To further support this hypothesis, we examined whether the treatment of patients with BD might affect the levels of circulating ECS. To this end, we determined serum ECS in patients with BD, before and after treatment. Blood samples were collected from ten patients immediately after admission to the acute psychiatric unit of the Eitanim Psychiatric Hospital (Eitanim, Israel) because of a manic psychotic episode. All patients were women with an average age of 35.36 ± 12.96 (S.D) years and were initially treated with clothiapine, clonazepam, lorazepam, or a combination thereof. Mood stabilizers, such as lithium and/or antipsychotic drugs, were added after the first blood collection. A second blood sample was collected 2–3 weeks later, after the patients had stabilized and had been released from the acute inpatient unit or the hospital. Mood and behavior stabilization, manifesting as longer sleep periods, fewer grandiose delusions, and decreased irritability and talkativeness, were determined by the professional medical staff. The samples were extracted, and ECS concentrations were determined as described in the Materials and Methods (Section 4). ECS concentrations in the sera of the patients before and after treatment are shown in Figure 7. The levels of ECS decreased by an average of 362% after treatment. Furthermore, as shown in Figure 7B, of the ten patients examined, seven showed a decrease in serum ECS. Since the distributions of the concentration values were not normal and the sample size was small (ten pairs), a nonparametric test, the exact one-tailed Wilcoxon matched-pairs signed rank test, was used to evaluate the effect of the treatment. Serum ECS was statistically significantly lower after treatment of the patients (Figure 7).

## 3. Discussion

In the past decade, we and others have presented evidence of the involvement of endogenous Na^+^, K^+^-ATPase inhibitors in BD [20,33]. We have shown that decreasing ECS in rats and Black Swiss and BALB/c male mice through i.c.v. administration of anti-Ou-Ab elicits anti-depression and anti-manic like behaviors [26,30]. These studies have suggested that the interaction of ECS with the α subunit of the Na^+^, K^+^-ATPase is involved in behavior and specifically may participate in BD symptoms. Therefore, we sought to decipher which of the three α isoforms of the Na^+^, K^+^-ATPase in the brain are involved in the ECS-induced alterations in behavior. We addressed this question experimentally by examining behavioral changes in depression, anxiety, and exploration behaviors in mouse models (FST and OFT after AMPH, respectively) after anti-Ou-Ab administration into the brain in α1^+/−^ and wt mice. Since the α2 isoform is concentrated primarily in glial cells, and α3 is found primarily in neuronal cells [34,35], performing the described experiments on α2^+/−^ and α3^+/−^ mice would have been of great interest. However, owing to the low fertility and high mortality of the offspring, we were unable to raise a sufficiently large colony of α2^+/−^ and α3^+/−^ mice to perform experiments, and those mice were not included in this study.

The behavior of α1, α2, and α3 haploinsufficient Black Swiss mice has been compared with that of wt mice [36,37], and α2^+/−^ mice have been found to display elevated anxiety-associated behavior, diminished locomotor activity, and impaired spatial learning in the Morris water maze, whereas α3^+/−^ heterozygous mice display spatial learning and memory deficits, elevated locomotor activity and locomotor response to methamphetamine. In contrast, no significant differences in behavior have been observed between α1^+/−^ and wt mice [36]. In agreement with the above findings, we demonstrated that, under control conditions, the behavior of α1^+/−^ mice was similar to that of wt mice in all parameters of the FST and OFT (Figure 1, Figure 2, Figure 3 and Figure 4). The present experiments were performed in C57Bl/6 male and female mice. Different strains of mice have been established to exhibit different behavior characteristics [38]; thus, comparison of the behavior obtained in this study with previous studies on other strains is inappropriate.

Differences in behavior between wt and α1 haploinsufficient mice were detected after the administration of anti-Ou-Ab. Although this treatment did not affect the behavior of the wt mice in FST and OFT, it decreased the activity of α1^+/−^ mice in FST and affected exploratory and anxiety behaviors in the OFT (Figure 1, Figure 2, Figure 3 and Figure 4). A plausible interpretation of these results is that under decreased α1 Na^+^, K^+^-ATPase levels (α1^+/−^ mice), the diminished ECS in the brain (after treatment with anti-Ou-Ab) might have elicited changes in depressive- and manic-like behaviors. Hence, we conclude that the α1 isoform of the Na^+^, K^+^-ATPase may participate in the ECS-induced changes in behavior.

The ECS-induced behavioral changes may result from direct interaction with the α1 isoform or indirectly by affecting α2 and α3 activities; the reduction in ECS (after treatment with anti-Ou-Ab) may directly cause disinhibition of α1 isoform transport activity and change intracellular signaling, resulting in alterations in behavior. The α1 isoform in rodents is relatively insensitive to CS compared to the other isoforms [6]. However, while the α1 isoform is expressed ubiquitously in all cell types of the brain, the α2 isoform is predominantly expressed in glial cells and α3 is expressed mostly in neurons in the rat nervous system [39]. Hence, the relatively high abundance of the α1 isoform may underline the biological effects. Importantly, ECS is a large group of cardenolides, bufadienolides, and their derivatives, which possess different affinities to the three α isoforms [40,41]. It is not known which of these steroids is responsible for the behavioral changes described in this study. There is a possibility that the changes in behavior following reduction in ECS (treatment with anti-Ou-Ab) under reduced α1 levels (α1^+/−^ mice) are mediated by alterations in α2 and α3 transport and/or signaling activities. This warrants consideration.

Determination of ECS in the brain (measured as ouabain-like immunoreactivity) showed that the levels in α1^+/−^ are dramatically lower compared to those in wt mice (Figure 5). Similar results were obtained when comparing ECS levels in the blood. The levels in the blood of α1^+/−^ mice were 76% lower compared to those in wt (Figure 5B). These surprising results suggest that there is a crosstalk between the density of α1^+/−^ protein and ECS levels. Such crosstalk may be at the levels of the synthesis or degradation pathway of the steroids, which have not yet been elucidated.

The behavioral effects of treatment with anti-Ou-Ab in the FST test were accompanied by a decrease in ECS in the brain and a concomitant increase in Na^+^, K^+^-ATPase activity (Figure 5 and Figure 6). Since ECS bind the anti-Ou-Ab, the decrease in brain ECS was expected. Correspondingly, because ECS are potent inhibitors of Na^+^, K^+^-ATPase activity, increased activity of this enzyme was also expected under these conditions. Our results suggested that ECS-induced alterations in behavior may be mediated by increased Na^+^, K^+^-ATPase activity.

The α1 isoform of Na^+^, K^+^-ATPase is present in all eukaryotic cells and is considered to function as a housekeeping pump responsible for the maintenance of Na^+^ and K^+^ gradients across the plasma membrane [3,42]. Therefore, the involvement of this isoform in specific cellular pathways and disease was somewhat unexpected. However, previous studies have demonstrated that the α1 isoform may be involved in specific pathological states. Kaphzan and colleagues have demonstrated that chronic inhibition of the α1 isoform of the Na^+^, K^+^-ATPase reverses axon elongation in the hippocampus in a mouse model of Angelman syndrome, thereby suggesting its involvement in this disease [43,44]. A recent review has summarized results supporting the involvement of the α1 isoform of the Na^+^, K^+^-ATPase in conditions including primary aldosteronism, Charcot–Marie–Tooth disease, peripheral neuropathy, complex spastic paraplegia, and hypomagnesemia accompanied by seizures [45]. Evidently, the α1 isoform, beyond its housekeeping role, is involved in specific cellular functions whose malfunctioning may result in specific diseases.

The results of our animal experiments and previous studies prompted us to investigate possible changes in circulating ECS in patients with BD after treatment. We demonstrated that the treatment of patients with BD significantly decreased serum ECS (Figure 7). We speculate that this decrease in ECS might potentially be responsible for the improvement in the patients after the treatment, although we do not have direct experimental evidence supporting this possibility. Importantly, however, we have recently demonstrated, in a preliminary study, that the treatment of patients with BD with anti-digoxin antibodies (Digibind), which decreases ECS in the circulation, elicits significant clinical improvements in patients with BD [46].

In conclusion, this study demonstrated that the action of ECS on depressive- and manic-like behaviors in C57Bl/6 mice is mediated by the α1 isoform of the Na^+^, K^+^-ATPase, thus implicating this isoform in BD. This finding, together with the decrease in circulating ECS observed in patients with BD after treatment, further supports that the Na^+^, K^+^-ATPase/ECS system is involved in behavior and in the etiology of BD.

## 4. Materials and Methods

### 4.1. Serum from Patients with BD

The study on human serum samples was approved by the Helsinki committees of Eitanim and Hadassah Hospitals, Jerusalem. The diagnosis of a manic episode was performed by the treating psychiatrists and verified by a staff psychiatrist from the research team. Blood samples (10 mL) were collected from ten patients immediately after admission to the emergency department at the Eitanim Psychiatric Hospital (Eitanim, Israel). A second blood sample (10 mL) was collected 2–3 weeks later, after the patients had stabilized. Informed consent was obtained at the second blood withdrawal. Samples from patients who did not provide consent were discarded. Samples were centrifuged (4 °C, 3000× *g*, 15 min) to obtain sera, which were frozen and stored at −80 °C until use.

### 4.2. Animals and Behavioral Tests

All procedures were performed in accordance with the Israel Ministry of Health Regulations and were approved by The Hebrew University of Jerusalem Animal Care and Use Committee (protocol #MD-17-15298-4). Mice were housed at the Hebrew University Animal Facility in a temperature-controlled SPF facility (22 ± 2 °C), with a 12 h/12 h light–dark cycle and food and water provided ad libitum. The α1 isoform mutated mice were obtained from Dr. Hanoch Kaphzan (Haifa University, Israel). A full description of the development of the α1^+/−^, α2^+/−^ and α3^+/−^ specific Na^+^, K^+^-ATPase knockout mice has been published previously [47,48]. The α^+/−^ specific Na^+^, K^+^-ATPase α1 heterozygous knockout male mice (^+/−^) were bred with wt mice (+/+) to produce (+/+) controls and (^+/−^) haploinsufficient offspring in a 1:1 ratio. Genotyping was performed on DNA isolated from tail samples obtained at the age of 3 weeks. DNA was extracted from each sample with a DNeasy kit from Qiagen and a PCR assay as previously described [30]. Adult male and female mice 3–5 months of age, of each genotype, were used in the behavioral experiments. We had difficulty in establishing α2^+/−^ and α3^+/−^ colonies; therefore, those strains were not examined in this study.

The surgical procedure and behavioral tests were performed as previously described [30]. Briefly, mice were divided randomly into two groups: control and anti-Ou-Ab-treated. Anti-Ou-Ab (1 μg/kg in 2.5 μL of artificial cerebrospinal fluid) or rabbit IgG (Sigma-Aldrich, St. Louis, MO, USA) were administered by i.c.v. injection into the lateral ventricle under light isoflurane sedation, as previously described [30]. The rabbit anti-ouabain antibodies were prepared and purified on protein Sepharose CL-4B beads and columns as previously described. Hence, the whole IgG fraction was used in this study. Notably, the specificity of the fraction towards ouabain was previously shown by demonstrating that the addition of ouabain to the preparation rescues antibodies-induced biological responses (cell growth) [49]. For the OFT, mice were placed in a 50 × 50 cm open-field arena for 6 min. For the FST, mice were placed in a water tank, and their swimming was monitored for 6 min. Activities were monitored and quantified with an Ethovision XT (Noldus Information Technologies, Wageningen, The Netherlands) automated camera-based computer tracking system. The mice were then sacrificed, and the brains were dissected and frozen (−80 °C) for later analysis.

### 4.3. Extraction and Determination of Endogenous Cardiac Steroids

The extraction and determination of ECS (endogenous ouabain-like immune-reactivity) were conducted as previously described [15,30]. In brief, samples of mouse left cortex tissue were homogenized, and serum samples were extracted with 0.1% TFA and methanol (10 mL/g) in a 1:1 *v*/*v* ratio. The samples were then centrifuged (4°, 15 min, 28,000× *g*). After centrifugation, the clear supernatant was decanted and evaporated, and the dry residue was dissolved in 3 mL phosphate-buffered saline. Each sample was loaded onto a Sep-pak C-18 column (Agilent Technologies, Santa Clara, CA, USA), which was then washed with 10 mL DDW containing 0.1% TFA, and the bound steroids were eluted with 80% acetonitrile. The solvent was evaporated, and the residue was dissolved in phosphate-buffered saline. ECS were determined with enzyme-linked immunosorbent assays testing samples’ ability to inhibit the specific binding of rabbit antibodies to solid phase-bound ouabain.

### 4.4. ATPase Activity

Crude synaptosomal membrane fractions were prepared from mouse right cortex samples, as described previously; Na^+^, K^+^-ATPase activity in the synaptosomal fraction was determined according to the amount of inorganic phosphate released during incubation at 37 °C, as previously described [15]. In brief, 250 µL of a synaptosomal preparation was added to 500 µL reaction buffer (50 mM Tris base, 120 mM NaCl, 10 mM KCI, 4 mM MgCl_2_, pH 7.4) in the presence or absence of 1 mM ouabain. After a 20 min incubation, 10 μl of ATP (2.5 mM final concentration) was added, and the incubation proceeded for an additional hour. The reaction was stopped by the addition of 1 mL 16% trichloroacetic acid, and the tubes were placed on ice for 10 min. After vortex mixing, 200 µL was removed for determination of inorganic phosphate through a colorimetric method, as described previously [50].

### 4.5. Statistical Analyses

Exact one-tailed Wilcoxon matched-pairs signed rank tests were used to evaluate differences between pairs. An unpaired two-tailed T-test was used to evaluate differences between the AMPH- and saline-treated mice. Mouse behavior and the levels of ECS and brain Na^+^, K^+^-ATPase activity were analyzed with a multiple comparison two-way ANOVA. All values are expressed as mean ± SE, and all analyses were performed in GraphPad Prism v 7.03 (GraphPad Software, Inc., San Diego, CA, USA); *p* < 0.05 was considered significant.

## Data Availability

Data is contained within the article.

## Abbreviations

bipolar disorder (BD), sodium, potassium-dependent adenosine triphosphatase (Na^+^, K^+^-ATPase), anti-ouabain antibody (anti-Ou-Ab), endogenous cardiac steroids (ECS), amphetamine (AMPH), intracerebroventricular (i.c.v.), intraperitoneal (i.p.), forced swimming test (FST), open field test (OFT), wild type (wt).
